# Predischarge Postpartum Methicillin Resistant *Staphylococcus aureus* Infection and Group B Streptococcus Carriage at the Individual and Hospital Levels

**DOI:** 10.1155/2014/515646

**Published:** 2014-03-06

**Authors:** Andrea M. Parriott, Joelle M. Brown, Onyebuchi A. Arah

**Affiliations:** ^1^Department of Health Policy and Management, UCLA Fielding School of Public Health, 650 Charles E. Young Drive S., 31-269 CHS, Los Angeles, CA 90095, USA; ^2^Department of Epidemiology, UCLA Fielding School of Public Health, 650 Charles E. Young Drive S., 71-236 CHS, Los Angeles, CA 90095, USA; ^3^Department of Epidemiology and Biostatistics, University of California, San Francisco, School of Medicine, 50 Beale Street, San Francisco, CA 94105, USA; ^4^UCLA Center for Health Policy Research, 10960 Wilshire Boulevard No. 1550, Los Angeles, CA 90024, USA; ^5^Department of Public Health, Academic Medical Center, University of Amsterdam, Meibergdreef 9, 1100 DE Amsterdam, The Netherlands

## Abstract

*Background*. We sought to characterize the relationship between individual group B streptococcus (GBS) colonization and pre-discharge postpartum methicillin resistant *Staphylococcus aureus* (MRSA) infection in United States women delivering at term. We also sought to examine the association between hospital GBS colonization prevalence and MRSA infection. *Materials and Methods*. Data was from the Nationwide Inpatient Sample, a representative sample of United States community hospitals. Hierarchical regression models were used to estimate odds ratios adjusted for patient age, race, expected payer, and prepregnancy diabetes and hospital teaching status, urbanicity, ownership, size, and geographic region. We used multiple imputation for missing covariate data. *Results*. There were 3,136,595 deliveries and 462 cases of MRSA infection included in this study. The odds ratio for individual GBS colonization was 1.2 (95% confidence interval: 0.9 to 1.5). For a five-percent increase in the hospital prevalence of GBS colonization, the odds ratio was 0.9 (95% CI: 0.1 to 5.6). *Conclusions*. The odds ratio estimate for the association of hospital GBS prevalence with MRSA infection is too imprecise to make conclusions about its magnitude and direction. Barring major bias in our estimates, individual GBS carriage does not appear to be strongly associated with predischarge postpartum MRSA infection.

## 1. Introduction

*Staphylococcus aureus* is a gram positive coccus that can cause asymptomatic colonization, as well as a wide variety of infections in humans, including skin lesions, cellulitis, pneumonia, necrotizing fasciitis, wound infections, sepsis, meningitis, endocarditis, urinary tract infections, and toxic shock syndrome [1, 2]. Methicillin-resistant* Staphylococcus aureus* (MRSA) refers to* S. aureus* strains that have developed resistance to *β*-lactam antibiotics, including penicillins, cephalosporins, and carbapenems [3].

Group B streptococcus (GBS, also known as* Streptococcus agalactiae*) is thought to cause asymptomatic colonization in 10–30% of late-term pregnant women. Maternal colonization with GBS is a risk factor for early neonatal GBS disease, which may be fatal or cause permanent disability. Because of this risk, the Centers for Diseases Control and Prevention recommend that all pregnant women be screened for GBS colonization between 35 and 37 weeks of gestation and that women who are found to be carriers receive prophylactic intrapartum antibiotic treatment (IPA) with penicillin or ampicillin (both *β*-lactam antibiotics) [4].

There are two primary reasons to suspect that there may be a correlation between GBS colonization and MRSA infection. The first is the use of intrapartum antibiotic prophylaxis, which is given to an estimated 87% of women who test positive for GBS prenatally [10]. While antibiotic exposure is an established risk factor for MRSA infection [11], the question of whether or not IPA causes antibiotic resistance is a controversial one. While most isolates of GBS are sensitive to penicillin and ampicillin, isolates with increased minimum inhibitory concentrations and in vitro resistance have been reported [4]. Aside from a single hospital based case-control study (described below) we could find no studies that examined whether women who have received IPA are at greater risk of subsequent drug resistant infections. However, several studies have found an association between IPA and neonatal infections with drug resistant organisms [12–14].

The second reason to suspect an association between GBS colonization and postpartum MRSA infection is that GBS colonized women may be more likely to be colonized with MRSA. A number of studies have investigated the association between rectovaginal carriage of GBS and* S. aureus* or MRSA carriage. Two studies reported increased odds of* S. aureus *colonization, and one study found increased odds of MRSA carriage in GBS positive women [5, 6, 15]. Another study found increased odds of GBS carriage in women colonized with MSSA relative to those with no* S. aureus *carriage and decreased odds of GBS carriage in women colonized with MRSA relative to those colonized with MSSA but could not detect an association between GBS and MRSA using women not colonized with* S. aureus* as the reference group [8]. For more details on the literature regarding GBS and MRSA carriage in pregnant women, see Table 1.

We could find only one study that examined the relationship between GBS carriage and invasive MRSA infection. A case control study of an outbreak of MRSA skin and soft tissue infections in a New York city hospital found no GBS colonization among 8 cases, while 11 of 46 controls were colonized (*P* = 0.03) [16]. That study also found that cases were less likely to have received IPA than controls (although the association was not significant at the *α* = 0.05 level). However, this study was limited to a single outbreak in one hospital and may not be representative of postpartum MRSA infection nationwide.

We sought to examine the relationship between GBS colonization and MRSA infection prior to discharge in US maternity inpatients delivering at term. We also sought to examine the association between hospital GBS colonization prevalence and predischarge MRSA infection. If an association between GBS carriage and invasive MRSA infections exists, the clinical implications would be dependent on the mechanism. If the association is due to increased prevalence of MRSA colonization in GBS carriers, it may be prudent to consider the costs and benefits of targeted MRSA screening and decolonization in GBS positive women. If the association is due to the use of IPA prophylaxis, then the risk of MRSA infections in the mother should be weighed against the benefits to the infant.

## 2. Materials and Methods

### 2.1. Data Source

The Nationwide Inpatient Sample (NIS) is a representative sample of approximately 20% of US community hospitals. Among hospitals included in the NIS, all inpatient discharges are reported. The NIS contains both hospital and patient level data. For patient level data, there is one record for each inpatient admission. Thus, one individual may contribute to multiple observations. The NIS is administered by the Healthcare Cost and Utilization Project (HCUP), a Federal-State-Industry partnership sponsored by the Agency for Healthcare Research and Quality (AHRQ). A complete list of agencies that contribute data to HCUP can be found at http://www.hcup-us.ahrq.gov/partners.jsp?NIS.

Because this study used a preexisting, deidentified, publicly available dataset, it was exempt from review by the Institutional Review Board of the University of California, Los Angeles.

### 2.2. Study Group

For this analysis, we included women in the NIS for years 2005 through 2008 who were admitted for delivery of an infant (Diagnosis Related Group 24 370–375) to a hospital with more than 50 deliveries per quarter. We excluded women with documented preterm delivery or inadequate prenatal care to minimize the number of women who had not been screened for GBS colonization.

### 2.3. Outcome Assessment

The outcome of interest was invasive MRSA infection prior to discharge after hospitalization for the delivery of an infant. In 2008, several new ICD-9 CM codes indicating MRSA infection or carriage were introduced including 038.12 (MRSA septicemia), 482.42 (methicillin resistant pneumonia due to* S. aureus*), and 041.12 (MRSA in conditions classified elsewhere and of unknown site), which are used to define invasive MRSA infections in 2008 admissions. Prior to 2008, invasive MRSA infection is defined by presence of ICD-9 codes 482.41 (*S. aureus* pneumonia), 038.11 (*S. aureus *septicemia), or 041.11 (*S. aureus* in conditions classified elsewhere and of unknown site) along with code V09.0 (infection with microorganisms resistant to penicillins).

### 2.4. Exposure Assessment

The primary exposure of interest in this analysis is carriage of or infection with GBS. Carriers of GBS were identified using ICD-9 CM codes 041.02 (group B streptococcal infection) and V02.51 (carrier or suspected carrier of group B streptococcus).

Due to the communicable nature of MRSA and its propensity to spread in hospital settings, the exposure status of other women in the same hospital may also put patients at risk. Therefore, the prevalence of GBS carriage in delivering women was also calculated for each hospital.

### 2.5. Covariates

Patient level covariates were race, age, expected payer (used as a proxy of socioeconomic status), and prepregnancy diabetes.

Hospital level variables used to create the sampling frame for the NIS were also included in our models. These variables were teaching status, urbanicity, ownership, size, and geographic region. Definitions of hospital size, ownership, and regions can be found in the* Introduction to the HCUP Nationwide Inpatient Sample*, at http://www.hcup-us.ahrq.gov/db/nation/nis/NIS_Introduction_2008.jsp [17].

### 2.6. Statistical Analysis

To estimate the association between GBS status and MRSA infection, we analyzed the data using hierarchical logistic regression with a random intercept.

Multiple imputation of missing variables was conducted using PROC MI in SAS version 9.2. Individual level covariates were imputed using a model that contained an indicator variable for the hospital. Hospital level variables were imputed at the hospital level, such that all patients in a given hospital have the same imputed value within an imputation. For comparison, the analysis was repeated without imputation.

Analyses were done using Statistical Analysis Software (SAS) version 9.2 and Stata version 12.

### 2.7. Bias Analysis

Obesity appears to be a risk factor for postpartum infection and MRSA skin and soft tissue infections [18–20]. GBS colonization may be more common in obese women; Stapleton et al. found a modest increased risk of GBS colonization with increasing BMI [21].

While the NIS contains a variable for obesity, only 2.2% of women in the sample were recorded as being obese. In contrast, 30% of women aged 20 to 39 in the 2005-2006 National Health and Nutrition Examination Survey and 18% of women aged 18 to 42 in the 2009 California Health Interview Survey were obese [22, 23]. This led us to suspect that the obesity measure in the NIS is unreliable and inadequate to control for possible confounding by obesity.

Probabilistic bias analysis for confounding by obesity was conducted by directly adjusting calculated measures of association, using the technique detailed by Lash et al. [24]. Five thousand iterations were run. The prior for the odds ratio for the effect of obesity on MRSA infection is lognormal, with 95% prior limits at 1.0 and 3.0. The prior for the proportion of obesity women among GBS colonized women is beta distributed, with *α* = 10.47 and *β* = 41.87. This corresponds to a unimodal distribution with a mean of 0.2 and 95% prior limits of 1% and 32%. The prior for the proportion of obese among GBS negative women is beta distributed with *α* = 1.76 and *β* = 9.99, corresponding to a unimodal distribution with a mean of 0.15 and 95% prior limits of 2% and 39%.

Due to recentering and rescaling of hospital GBS prevalence, the odds ratio compares the odds of MRSA infection in a hospital with a prevalence of 20% to the odds of infection in a hospital with a prevalence of 15%. To create expected prevalence of obesity for the “exposed” and “unexposed” group, we took a weighted average of the numbers drawn from the beta distributions for obesity prevalence among GBS positive and GBS negative women. For 15% hospital GBS prevalence, the value drawn from the GBS negative distribution was assigned a weight of 0.85, and the value drawn from the GBS positive distribution was assigned a weight of 0.15. For 20% hospital prevalence, the weights were 0.8 for the GBS negative distribution and 0.2 for the GBS positive distribution.

## 3. Results

A total of 3,136,595 women with no record of preterm delivery or inadequate prenatal care delivered infants in hospitals with 50 or more deliveries per quarter. Among these women, there were 500,225 documented cases of GBS colonization (16%) and 462 documented cases of MRSA infection.  Table 2 displays the univariate distributions of the outcome and covariates by GBS colonization status.

We were unable to determine the site of MRSA infection in just over half of cases. The most common documented sites of infection were skin (32% of infections), urinary tract (7%), other genitourinary sites (5%), and wounds (3%).

The results of the multivariable analysis, including bias adjustment for unmeasured confounding by obesity, are given in Table 3. The adjusted odds ratio for individual GBS colonization was 1.2, and the 95% confidence interval was 0.9 to 1.5. The OR for hospital GBS colonization prevalence was 0.9, and the 95% CI was 0.1 to 5.6. For comparison, a model was run without imputation of missing values, using only complete records. The change in the odds ratio and confidence interval for individual colonization was negligible while the odds ratio for hospital GBS prevalence was 1.3, and the 95% confidence limit was 0.2 to 11.3.

In the bias analysis, the mean value of the bias factor (the ratio of the biased to the adjusted odds ratio) for individual GBS carriage was 1.05, and the variance of the log bias factor was 0.01. The bias adjusted median odds ratio was 1.1, and the 95% simulation interval was 0.8–1.5. For the odds ratio for hospital GBS prevalence, the distribution of the bias control factor had a mean very nearly equal to one (1.003) and a variance very nearly equal to zero (3.2 × 10^−5^); thus the bias adjusted odds ratio and 95% simulations interval are equal to the unadjusted estimate and 95% confidence interval.

## 4. Discussion

While other studies (noted above) have investigated the relationship between GBS carriage and MRSA colonization in pregnant and laboring women, we could identify no other studies that examined the risk of invasive MRSA infection in a nationally representative sample. Thus, our results cannot be interpreted in the context of previous work, making it difficult to draw comparative conclusions. Before considering the amount of evidence this study provides, several limitations should be considered.

A major weakness of this study is that followup is limited to the predischarge period, as the NIS only includes information on diagnoses that occurred prior to discharge after delivery of an infant and contains no mechanism to track patients who are readmitted. Because the predictors of predischarge infection may not be representative of the predictors of postpartum MRSA as a whole, these findings should not be generalized to the postdischarge period. Recognition of this limitation of generalizability is important, since the majority of postpartum infections are diagnosed after hospital discharge [25].

There is also potential for misclassification of the outcome variable. Diagnosis of MRSA infection typically requires antibiotic susceptibility testing, which may not be performed in some cases, particularly when an infection resolves spontaneously. It is also possible that diagnosed MRSA infections may not be correctly coded in discharge data. Schweizer et al. conducted a validation study of the use of discharge data to detect MRSA infections, using medical record review as the gold standard. They found excellent specificity (99%) but low sensitivity (20%) [26].

An additional limitation of this analysis is the possibility of incomplete reporting of the exposure variable. The reported prevalence of GBS carriage in delivering women who do not have diagnostic codes for premature labor or inadequate prenatal care is 16%. This falls within the CDC's reported prevalence of 10% to 30% [4]. However, the NIS is constructed from data gathered for the purposes of hospital billing, and discharge data may underreport diagnoses and conditions that are not likely to result in additional compensation for the hospital. It is also possible that inadequate prenatal care and preterm birth (particularly late preterm birth, where management of the labor and the neonate differs a little from term birth) are underreported. We could find no validation studies of the use of administrative data to determine GBS colonization or infection status. However, validation of hospital discharge data for other pregnancy conditions has generally shown very high specificity, but sometimes low sensitivity [27].

As we expect the specificity of our exposure and outcome variable to be excellent, we would not expect bias from misclassification as long as errors in exposure and outcome status were nondifferential and uncorrelated [28]. However, we cannot rule out the possibility of differential misclassification. Of particular concern is the possibility of positive correlation of exposure and outcome misclassification. Table S1 (see Supplementary Material available online at http://dx.doi.org/10.1155/2014/515646) shows the possible values of the odds ratio under different values of sensitivity of the outcome, given the recorded exposure status.

The limitations discussed above could be substantially reduced by examining the relationship between GBS colonization and MRSA infection in a setting where information from postpartum readmissions and outpatient care (particularly prenatal care visits) is available. This would allow extension of the follow-up period beyond the hospitalization for delivery and permit restriction of the study group to women who are known to have been screened for GBS colonization. A staff model health maintenance organization (HMO), where patients receive nearly all of their care from a centralized organization, would be an ideal setting for further examination of these research questions.

The primary strength of this analysis lies in the large sample size, which provides adequate power to examine the rare outcome of postpartum MRSA infection. The sample is also designed to be representative of the population of US nonfederal hospitals. As all patients within participating hospitals were sampled, there is no potential for selection bias at the patient level.

## 5. Conclusion

This analysis did not detect an association between either GBS colonization at the hospital level or hospital prevalence of GBS colonization and predischarge MRSA infection in women admitted for delivery of an infant. Furthermore, given the assumptions of our data model (including absence of any major source of bias) our data are compatible with, at most, a modest association between individual GBS carriage and risk of invasive MRSA infection.

The 95% confidence interval for the association of hospital GBS prevalence and predischarge MRSA infection is very wide (0.1 to 5.6). While we were unable to detect a relationship, our results are too imprecise to rule out large effects in either direction, even if we assume no uncontrolled bias.

## Supplementary Material

This table presents a sensitivity analysis for the possible effects of differential documentation of MRSA infection on the odds ratio for the effect of individual GBS colonization on pre-discharge postpartum MRSA infection. Expected values of the odds ratio are given for specified values of sensitivity of MRSA documentation in GBS positive and GBS negative women.

## Figures and Tables

**Table 1:** Associations between group B streptococcus colonization and *S*. *aureus* colonization in pregnant women.

Outcome	Study	OR	95% confidence interval
All *S. aureus *	Andrews et al., 2008 [5]	1.6	1.4–1.9
	Chen et al., 2006 [6]	2.1	1.7–2.5
	Creech et al., 2010 [7]	NR	Includes 1

MRSA	Andrews et al., 2008 [5]	2.2	1.6–1.8
	Chen et al., 2007 [8]	0.1*	0.0–0.7
		0.4**	0.0–3.5
	Creech et al., 2010 [7]	NR	Includes 1
	Reusch et al., 2008 [9]	0.6	0.1–5.0

MSSA	Chen et al., 2007 [8]	4.5	1.7–11.0

NR: not reported; *reference group is MSSA positive women; **reference group is *S. aureus* negative women, crude OR.

**Table 2:** Patient and hospital characteristics by GBS status and prevalence.

Variable	Category	Patient GBS status		Hospital GBS prevalence	
		Noncarrier	Carrier	≤15%	>15%
Total number		2,636,370	500,225	1,341,521	3,136,595
MRSA infection (%)		0.01	0.02	0.02	0.01
Race* (%)	White	39.04	40.12	35.69	41.85
	Black	8.20	12.02	7.67	9.66
	Hispanic	19.58	14.61	26.71	12.86
	Asian	3.87	3.92	3.70	4.01
	Other	4.29	3.97	4.23	4.24
Age* (%)	<20	9.81	10.06	11.60	9.26
	20–29	52.29	51.95	54.31	50.71
	30–39	35.12	35.44	31.55	37.09
	40+	2.67	2.54	2.43	2.89
Expected payer* (%)	Private insurance	51.93	55.39	47.65	57.21
	Medicaid	40.98	38.63	44.03	36.59
	Other payer	6.93	5.85	8.15	5.73
Diabetes (%)		0.72	0.77	0.71	0.74
Teaching hospital (%)		45.24	48.25	40.48	49.64
Urban hospital (%)		89.20	90.53	86.80	91.36
Hospital size^∗†^ (%)	Small	11.69	12.06	11.14	12.20
	Medium	25.76	25.43	27.35	24.48
	Large	62.42	62.39	61.35	63.22
Hospital control^†^ (%)	Private or public, collapsed	57.21	60.52	51.61	62.32
	Public	7.05	6.61	7.44	6.63
	Private, nonprofit	21.24	20.37	22.18	20.29
	Private, investor owned	10.72	9.33	13.74	8.08
	Private, collapsed	3.66	3.04	4.87	2.58
Hospital region (%)	Northeast	16.39	17.67	13.42	18.97
	Midwest	20.47	21.49	18.03	22.57
	South	37.81	37.11	38.69	36.95
	West	25.33	23.73	29.85	21.51

*Percentages may not add to 100 due to missing values.

^†^See *Introduction to the HCUP Nationwide Inpatient Sample* for an explanation of variable categories.

**Table 3:** Multivariable adjusted* odds ratio and 95% confidence intervals for the association of individual GBS carriage and hospital GBS prevalence with early postpartum MRSA infection.

Analysis	Exposure	Odds ratio	95% CI
Without imputation of missing values	Individual GBS colonization	1.2	0.9–1.5
	Hospital prevalence of GBS colonization**	1.3	0.2–11.3
With multiple imputation of missing values	Individual GBS colonization	1.2	0.9–1.5
	Hospital prevalence of GBS colonization**	0.9	0.1–5.6
With multiple imputation and bias adjustment for obesity	Individual GBS colonization	1.1	0.8–1.5
	Hospital prevalence of GBS colonization**	0.9	0.1–5.6

*All models adjusted for patient age, race, expected payer, and prepregnancy diabetes and hospital teaching status, urbanicity, bed size, ownership, and geographic region.

**Odds ratio is for a 5% increase in the prevalence of GBS colonization.

## References

[1] Heymann DL (2008). Staphyloccocal diseases. *Control of Communicable Diseases Manual*.

[2] Grundmann H, Aires-de-Sousa M, Boyce J, Tiemersma E (2006). Emergence and resurgence of meticillin-resistant *Staphylococcus aureus* as a public-health threat. *The Lancet*.

[3] Chambers HF, DeLeo FR (2009). Waves of resistance: *Staphylococcus aureus* in the antibiotic era. *Nature Reviews Microbiology*.

[4] Verani JR, McGee L, Schrag SJ (2010). Prevention of perinatal group B streptococcal disease—revised guidelines from CDC, 2010. *Morbidity and Mortality Weekly Report*.

[5] Andrews WW, Schelonka R, Waites K, Stamm A, Cliver SP, Moser S (2008). Genital tract methicillin-resistant *Staphylococcus aureus*: risk of vertical transmission in pregnant women. *Obstetrics and Gynecology*.

[6] Chen KT, Huard RC, Della-Latta P, Saiman L (2006). Prevalence of methicillin-sensitive and methicillin-resistant *Staphylococcus aureus* in pregnant women. *Obstetrics and Gynecology*.

[7] Creech CB, Litzner B, Talbot TR, Schaffner W (2010). Frequency of detection of methicillin-resistant *Staphylococcus aureus* from rectovaginal swabs in pregnant women. *American Journal of Infection Control*.

[8] Chen KT, Campbell H, Borrell LN, Huard RC, Saiman L, Della-Latta P (2007). Predictors and outcomes for pregnant women with vaginal-rectal carriage of community-associated methicillin-resistant *Staphylococcus aureus*. *American Journal of Perinatology*.

[9] Reusch M, Ghosh P, Ham C, Klotchko A, Singapuri S, Everett G (2008). Prevalence of MRSA colonization in peripartum mothers and their newborn infants. *Scandinavian Journal of Infectious Diseases*.

[10] van Dyke MK, Phares CR, Lynfield R (2009). Evaluation of universal antenatal screening for group B streptococcus. *The New England Journal of Medicine*.

[11] Tacconelli E, de angelis G, Cataldo MA, Pozzi E, Cauda R (2008). Does antibiotic exposure increase the risk of methicillin-resistant *Staphylococcus aureus* (MRSA) isolation? A systematic review and meta-analysis. *Journal of Antimicrobial Chemotherapy*.

[12] Joseph TA, Pyati SP, Jacobs N (1998). Neonatal early-onset *Escherichia coli* disease: the effect of intrapartum ampicillin. *Archives of Pediatrics and Adolescent Medicine*.

[13] Glasgow TS, Young PC, Wallin J (2005). Association of intrapartum antibiotic exposure and late-onset serious bacterial infections in infants. *Pediatrics*.

[14] Stoll BJ, Hansen N, Fanaroff AA (2002). Changes in pathogens causing early-onset sepsis in very-low-birth-weight infants. *The New England Journal of Medicine*.

[15] Top KA, Huard RC, Fox Z (2010). Trends in methicillin-resistant *Staphylococcus aureus* anovaginal colonization in pregnant women in 2005 versus 2009. *Journal of Clinical Microbiology*.

[16] Saiman L, O’Keefe M, Graham PL (2003). Hospital transmission of community-acquired methicillin-resistant *Staphylococcus aureus* among postpartum womenn. *Clinical Infectious Diseases*.

[17] (2010). *Introduction to the HCUP Nationwide Inpatient Sample (NIS) 2008*.

[18] Sebire NJ, Jolly M, Harris JP (2001). Maternal obesity and pregnancy outcome: a study of 287,213 pregnancies in London. *International Journal of Obesity*.

[19] Falagas ME, Kompoti M (2006). Obesity and infection. *The Lancet Infectious Diseases*.

[20] Sreeramoju P, Porbandarwalla NS, Arango J (2011). Recurrent skin and soft tissue infections due to methicillin-resistant *Staphylococcus aureus* requiring operative debridement. *American Journal of Surgery*.

[21] Stapleton RD, Kahn JM, Evans LE, Critchlow CW, Gardella CM (2005). Risk factors for group B streptococcal genitourinary tract colonization in pregnant women. *Obstetrics and Gynecology*.

[22] Ogden CL, Carroll MD, McDowell MA, Flegal KM (2007). Obesity among adults in the United States—no statistically significant chance since 2003-2004. *NCHS Data Brief*.

[23] Ask CHIS UCLA Center for Health Policy Research. http://ask.chis.ucla.edu/main/default.asp.

[24] Lash TL, Schmidt M, Jensen AØ, Engebjerg MC (2010). Methods to apply probabilistic bias analysis to summary estimates of association. *Pharmacoepidemiology and Drug Safety*.

[25] Yokoe DS, Christiansen CL, Johnson R (2001). Epidemiology of and surveillance for postpartum infections. *Emerging Infectious Diseases*.

[26] Schweizer ML, Eber MR, Laxminarayan R (2011). Validity of ICD-9-CM coding for identifying incident Methicillin-Resistant *Staphylococcus aureus* (MRSA) infections: is MRSA infection coded as a chronic disease?. *Infection Control and Hospital Epidemiology*.

[27] Lydon-Rochelle MT, Holt VL, Cárdenas V (2005). The reporting of pre-existing maternal medical conditions and complications of pregnancy on birth certificates and in hospital discharge data. *American Journal of Obstetrics and Gynecology*.

[28] Copeland KT, Checkoway H, McMichael AJ, Holbrook RH (1977). Bias due to misclassification in the estimation of relative risk. *American Journal of Epidemiology*.

