# Supplementary material for: Predischarge Postpartum Methicillin Resistant Staphylococcus aureus Infection and Group B Streptococcus Carriage at the Individual and Hospital Levels
Source: Infect Dis Obstet Gynecol. 2014 Mar 6;2014:515646. doi: 10.1155/2014/515646 (PMC3963373; doi:10.1155/2014/515646)
Supplement: Supplementary file 1 — This table presents a sensitivity analysis for the possible effects of differential documentation of MRSA infection on the odds ratio for the effect of individual GBS colonization on pre-discharge postpartum MRSA infection. Expected values of the odds ratio are given for specified values of sensitivity of MRSA documentation in GBS positive and GBS negative women. [file 515646.f1.pdf]

Table S1: ***Odds ratios for the effect of GBS colonization on early postpartum MRSA infection under different values of sensitivity of MRSA documentation***

		Sensitivity in GBS+		
		10%	20%	30%
Sensitivity in GBS-	10%	1.1	0.6	0.4
	20%	2.2	1.1	0.7
	30%	3.3	1.7	1.1
